# Observations on the Utility of Hedera Sylvestris (Ground-Ivy) in the Treatment of Mania

**Published:** 1819-09

**Authors:** E. Sutliffe


FOR THE LONDON MEDICAL AND PHYSICAL JOURNAL.

### *Observations on the Utility of Hedera Sylvestris (Ground-Ivy) in the Treatment of Mania.* By E. SUTLIFFE, Esq.

THE medical qualities of ground-ivy are not, I believe, sufficiently known to the medical profession, (for it is not admitted into our Pharmacopœia.) I have employed it for more than twenty years in cases of mental aberration, especially in melancholia, with the most advantageous results, and am disposed to think that it is a highly valuable remedy. It appears to act as a direct sedative. When its operation is salutary, it tranquillizes the patient; and when no organic disease is present, and the affection seems to depend on a casual cause of excitement, I am not often disappointed in my expectations of a favourable termination of the malady under its use.

The mode in which I have generally directed it to be employed, is a wine-glass full of the liquid juice twice or thrice a-day, but in some cases this cannot be exhibited; I have then ordered a proportionate quantity of the extract: but my observations have not led me to repose so full a degree of confidence in the latter as in the former preparation of the plant.

In cases of mania, where the high arterial excitement requires *local reduction*, I have found it, conjoined with abstraction of blood, generally lead the disease to a favourable issue. In some cases it has, however, not been productive of any benefit: in these, I suspect, there has been present some organic disease.

My object in making the preceding brief observations, is to draw the attention of other practitioners to this remedy, and to extend the benefits that may be derived from it beyond the circle of my own personal avocation.

*Queen-street, Cheapside; July 8th*, 1819.

